# Radioisotope Identification and Nonintrusive Depth Estimation of Localized Low-Level Radioactive Contaminants Using Bayesian Inference

**DOI:** 10.3390/s20010095

**Published:** 2019-12-23

**Authors:** Jinhwan Kim, Kyung Taek Lim, Kilyoung Ko, Eunbie Ko, Gyuseong Cho

**Affiliations:** Department of Nuclear & Quantum Engineering, Korea Advanced Institute of Science and Technology, 291 Daehak-ro, Yuseong-gu, Daejeon 34141, Korea; kjhwan0205@kaist.ac.kr (J.K.); kl2548@kaist.ac.kr (K.T.L.); coltom@kaist.ac.kr (K.K.); cutsky@kaist.ac.kr (E.K.)

**Keywords:** remote depth profiling, radioisotope identification, Bayesian inference, uncertainty estimation, gamma spectral analysis, low-level radioactive contaminants, nuclear decommissioning, low-resolution detector

## Abstract

Obtaining the in-depth information of radioactive contaminants is crucial for determining the most cost-effective decommissioning strategy. The main limitations of a burial depth analysis lie in the assumptions that foreknowledge of buried radioisotopes present at the site is always available and that only a single radioisotope is present. We present an advanced depth estimation method using Bayesian inference, which does not rely on those assumptions. Thus, we identified low-level radioactive contaminants buried in a substance and then estimated their depths and activities. To evaluate the performance of the proposed method, several spectra were obtained using a 3 × 3 inch hand-held NaI (Tl) detector exposed to Cs-137, Co-60, Na-22, Am-241, Eu-152, and Eu-154 sources (less than 1μCi) that were buried in a sandbox at depths of up to 15 cm. The experimental results showed that this method is capable of correctly detecting not only a single but also multiple radioisotopes that are buried in sand. Furthermore, it can provide a good approximation of the burial depth and activity of the identified sources in terms of the mean and 95% credible interval in a single measurement. Lastly, we demonstrate that the proposed technique is rarely susceptible to short acquisition time and gain-shift effects.

## 1. Introduction

Sites near nuclear power plants are susceptible to large-scale land and building contamination because of the significant amount of radioactive waste generated by such facilities. It is, therefore, important to acquire information on the wastes present on these sites on behalf of project management and engineering services working on environmental restoration [1,2,3]. In particular, depth profiling of radioactive contaminants is critical for determining the most cost-effective decommissioning strategy, because the quantity of radioactive waste required for disposal can be reduced considerably by removing surface contamination at varying depths [4]. Nevertheless, the task of depth profiling is still difficult to achieve because porous materials such as soil and concrete covering the contaminants can act as a shield, resulting in the attenuation of emitted radiation.

One example of such waste is on the beaches of Dounreay in Northern Scotland, where radioactive soil contaminants are widely spread along the beach [5,6]. This is due to the so-called Dounreay hot particles that are mainly composed of Cs-137 and Co-60, released from the fuel processing of the Material Test Reactor at the Dounreay nuclear facility. Other examples of buried radioactive contaminants include orphan radioactive sources [7]. An orphan source is generally a sealed source of radioactive material that has been lost, abandoned, misplaced, stolen, or otherwise transferred without proper authorization [8].

Therefore, various non-destructive methods for remote-depth profiling have been reported in many papers [9,10,11,12,13,14,15,16,17,18]. However, the majority of the nonintrusive methods reported in these studies were based on a frequentist approach; that is, they required repeated measurements in order to provide a mean value with a standard error. Also, the maximum detectable depth of these methods was not sufficient to detect deeply buried contaminants. Therefore, a new approach, based on Bayesian inference, has recently been developed [19] to overcome the limitations imposed by older methods. This method can offer more reliable results because the output of burial depth analysis can be expressed as a probability distribution, even in a single measurement. In addition, its capability for maximum detectable depth for weak activity of the 0.94-μCi Cs-137 and 0.69-μCi Co-60 sources is superior in comparison with the existing methods. However, this method still assumes that only a single radioisotope is present in the substance and that no other radioisotopes will interfere with the measurement; a common assumption that is prevalent in other studies. But such assumptions can seriously undermine the results of a burial depth analysis in which there are different or multiple radioisotopes present.

Consequently, the objective of this study is first to identify all low-level radioactive contaminants buried in any substance, and then estimate remote depth profiling for the identified radioisotopes using Bayesian inference. In this study, radioactive sources of Cs-137, Co-60, Na-22, Am-241, Eu-152, and Eu-154, which are common elements encountered during decommissioning of nuclear facilities, were considered for the depth profiling. For convenience, the set of these radioisotopes will hereafter be referred to as the radioisotope library. Experimental results analyzed from various spectra, composed of not only single but also multiple radioisotopes, have been addressed to evaluate the performance of the proposed method. Furthermore, we have investigated the depth estimation performance of the proposed method in terms of data acquisition time and gain-shift effects due to calibration drift.

## 2. Materials and Methods

### 2.1. Bayesian Inference

Probability is one of the quantities that measure an event with an uncertainty that is associated with that particular event. There are two general philosophies providing different interpretations of probability: namely, frequentist inference and Bayesian inference [20]. In a frequentist approach, the probability is associated with the long-term frequency or proportion of events, in which the unknown parameters are treated as fixed values. That is, a frequentist does not associate probabilities with random variates, and only repeatable events can have probabilities in a statistical process. In contrast, a Bayesian approach is rooted in the belief that probabilities can be associated with unknown parameters (i.e., treated as random variables) to represent the uncertainty in any occurrence. That is, it can lead to much more intuitive results. For example, suppose you want to know the possibility that Korea will host the next World Cup. Bayesians are willing to assign a legitimate probability to Korea hosting the next World Cup based on the degrees of belief on the possible outcomes and every available information. Unlike Bayesians, frequentists do not assign any numerical probability to the same event because the World Cup cannot be regarded as a hypothetically repeatable process. This is a philosophical issue that frequentists can run into [21]. Also, some of the resultant interpretations are not particularly intuitive.

A Bayesian inference determines the probability distribution over the parameter or equivalently, the posterior distribution p(θ|y) of random variables θ, given prior distributions p(θ), and likelihood function p(y|θ) by applying Bayes’ theorem:

(1) $$p\left(\theta |y\right)=\frac{p\left(y|\theta \right)\;p\left(\theta \right)}{p\left(y\right)},$$

In the past, the challenge of applying the Bayesian inference to real-field applications was mainly around the computation requirement for the intractable high-dimensional integrals in the evidence p(y). However, it is now possible, owing to recent advances made in computation technology and in marginal-estimation techniques. The Markov Chain Monte Carlo (MCMC) algorithm is a technique that is widely used for approximate inference, in which the posterior distribution is estimated through a collection of samples via the Markov process [22]. Since the late 1940s, there has been tremendous progress in the field of statistics, seeing the development of such techniques as the Metropolis Hasting algorithm, the Hamiltonian Monte Carlo, and more recently, the No-U-Turn sampler [23]. These algorithms were based on MCMC so that they could obtain the posterior probability of parameters with accuracy. However, their relatively high costs in computation and their inefficient processes have hindered their usage in real-world applications. An alternative method that can overcome these limitations is to convert the computation of p(θ|y) to an optimization problem, also known as variational inference.

With variational inference, we assume there is a parameterized family of distributions q(θ;υ) (or equivalently, a variational distribution); then, we find the setting of the parameters that minimize the Kullback-Leibler (KL) divergence to the posterior distribution of interest:

(2) $$\upsilon^{*}=argminKL\left(q\left(\theta ;\upsilon \right)||p\left(\theta |y\right)\right).$$

The optimized $q\left(\theta ;\upsilon^{*}\right)$ is then regarded as an approximation to the posterior distribution. Since the KL divergence involving the posterior distributions lacks an analytic form, we instead maximize the evidence lower bound (ELBO):

(3) $$ℒ\left(\upsilon \right)=E_{q}\left[logp\left(\theta ,y\right)\right]-E_{q}\left[logq\left(\theta ;\upsilon \right)\right].$$

This can be simplified further by taking a mean-field approximation, where the parameters in the variational family are assumed to be fully factorized to independent variables. However, the difficulties arising from the model-specific derivations and implementations in developing such algorithms still hinder its use in practical applications. However, automatic differentiation variational inference (ADVI), which is a gradient-based method, can resolve this complexity in computation by providing a recipe for an automatic solution based on variational inference [24]. The underlying idea of ADVI is to transform the space of latent variables and to automate derivatives of the joint distribution by relying on the capabilities of probabilistic programming systems. For programming ADVI computation, we used Python language with the probabilistic programming framework of PyMC3 to establish a probability model and execute variational inference.

### 2.2. Model Specification

By defining a mathematical model that describes an observed spectrum in terms of the burial depth, activity, and shift degree of the spectrum, we can identify buried radioisotopes and obtain the posterior distribution of the depth and activity of the identified sources. Such a model can be established by extending the model defined by Kim et al. [19,25]

(4) $$M_{i}=\overset{J}{\underset{j=1}{\sum }}\frac{A_{j}P_{j}\delta_{j}}{4\pi h^{2}}e^{-\mu_{A}h}f\left(z_{j},\;\eta i\right)+cB_{i}\;for\;i=1,\ldots ,K$$

Here, $M_{i\;}$ is the measured spectrum (s^−1^) with i representing the channel (0<i≤K); J is the total number of radioisotopes; $A_{j}$ is the activity of the radioisotope (μCi); $P_{j}\;$is the total sum of gamma emission probabilities within the energy range of interest (i.e., 2.8 $\gamma s^{-1}Bq^{-1}$ for the 511 and 1275 keV gamma rays of Na-22); $\mu_{A}$ is the linear attenuation coefficient of gamma-ray in air (cm^−1^); *h* is the detection height measured from the detector to the surface of a given material (cm); z is the buried depth of a radioactive source (0≤z≤D cm) in a material from the front surface; η is the shift degree of the spectrum; $B_{i}$ is the background spectrum measured for *K* channels with c being its proportionality constant; δ is the effective front area (cm^−1^); and f(z, ηi) is the bilinear interpolation function. Computation of f(z, ηi) requires a spectrum measurement at depths ranging from 0 to D cm at certain intervals to determine the K×D response matrix for a radioisotope. Consequently, f(z, ηi) can be interpolated using the closest points to the f(z, ηi) among the known values of depths and channels from the K×D response matrix [19]. The parameter δ can be obtained experimentally by placing a source on the material surface (that is, at 0 cm depth), which can be expressed as:

(5) $$\delta =\frac{4\pi r^{2}N}{APe^{-\mu_{A}r}},\;$$

where N is the total net counts of the spectrum (s^−1^), and r is the detection height (cm) between the detector and the surface of a material.

Thus, the proposed model defines the function f(z,A, η, c) where the variable marked in bold type represents a vector notation. In practice, the existence of inevitable uncertainties inherent to the physical processes, such as radioactive disintegration, has an effect on the measured spectrum. In this regard, we can assume that the spectrum is normally distributed with a zero mean and variance of $\sigma^{2}$

(6) $$P\left(M|z,A,\;\eta ,\;c\right)=N\left(f\left(z,A,\;\eta ,\;c\right),\;\sigma^{2}\right).$$

The availability of prior distributions for z,  A,  η,  c, and$,\sigma^{2}$, which represents our knowledge of the parameters before taking any measurements, is assumed by Kim et al. [19]. That is, $A,c,\;\mathrm{and}\;\sigma^{2}$ followed gamma distributions with parameters (1, 1); z and η followed uniform distributions with parameters (0, 18) and (0.85, 1.15), respectively. These prior distributions reflected our belief that the sources might be buried less than 18 cm in the sand, and their activities would be low.

### 2.3. Procedures on Spectral Analysis

The spectral analysis of the depth estimation is a two-step process. First, the radioisotopes that are least likely to have generated an observed spectrum are excluded according to certain criteria [25]. This step is necessary because the model assigns a probability distribution to the parameters of every radioisotope present in the radioisotope library. For instance, the ratio of the standard deviation$\;\sigma_{j}$ to the mean$\;u_{j}$ of a radioisotope, i.e., relative standard deviation (RSD), can have a large value where a certain radioisotope in the library is not contributing to the spectrum. In terms of the magnitude of RSD, a small value suggests that the data are clustered tightly around the mean while the opposite is true in a large value of RSD. In addition, a radioisotope that does not attribute to the spectrum may have a relatively negligible contribution. The relative contribution (RC) of the radioisotope, $C_{j}$ to the spectrum can be expressed as:

(7) $$C_{j}=\frac{\frac{A_{j}P_{j}}{z_{j}{}^{2}}}{\sum_{j=1}^{J}\frac{A_{j}P_{j}}{z_{j}{}^{2}}}.$$

Here, radioisotopes can be regarded as present when the following conditions are satisfied:

(8) $$C_{j}>3\%\;\mathrm{and}\;\frac{\sigma_{i}}{u_{i}}\;<\;0.2.$$

These thresholds are subject to change depending on the situation. The first analysis can be thought as the identification step. Second, the identified radioisotopes through the first analysis are analyzed to obtain their final depths and activities.

### 2.4. Experimental Setup

**Figure 1:**
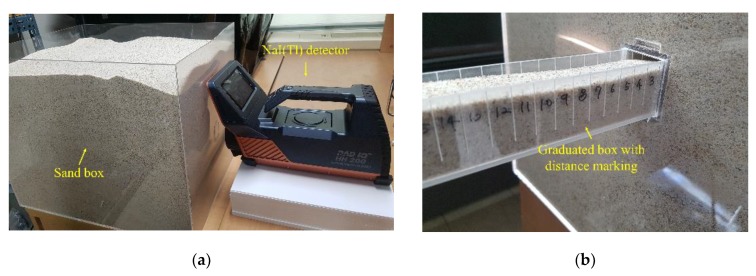
(**a**) An acrylic box filled with sand and an NaI (Tl) detector for gamma spectroscopy; and (**b**) a graduated box marked with the buried distance of the source measured from the front surface of the sandbox.

Gamma-ray spectra were obtained on radioactive sources buried in a sandbox filled with fine silica sand by using a 3 × 3 inch hand-held NaI (Tl) detector (NUCARE, Rad IQ^TM^ HH200, Incheon, Korea) that was located 3 cm away from the surface of the box, as depicted in Figure 1a. The detector was used only for the purpose of acquiring gamma spectra and the recorded raw data were then processed and analyzed separately through Python. The sandbox was composed of 0.3 cm-thick acrylic sheet forming a tank of 50 cm × 40 cm × 40 cm (length × width × height). The thickness of the acrylic sheets was chosen so that the gamma rays emitting from the source would be scattered in the sand matrix. The activities of the sources used for the experiments were 0.94 μCi, 0.69 μCi, 0.50 μCi, 0.90 μCi, 0.89 μCi, and 0.84 μCi for Cs-137, Co-60, Na-22 Am-241, Eu-152, and Eu-154, respectively. In addition, the sources were buried in a graduated box (50 cm × 0.3 cm × 0.3 cm) that was inserted into the main box to position the sources at the exact location in relation to the front of the sandbox surface, as shown in Figure 1b.

The response matrix was obtained by placing the sources in the graduated sandbox at depths of 0 cm, 3 cm, 7 cm, 12 cm, and 18 cm. Then, the spectra were measured at each depth for 30 min to ensure that minimal statistical fluctuation was achieved. A background spectrum for the response matrix was obtained under identical conditions in the absence of sources. For the energy range of spectra, values were chosen from 20 to 1600 keV (i.e., 563 channels). During these experiments, energy calibration was performed prior to taking each measurement via the built-in automatic calibration function provided by the detector system. This function is based on the energy emitted by the primordial radioisotope of K-40 (1461 keV). The automatic calibration function was not used for the acquisition of the test spectra because this method automatically compensates the gain-shift effects because of changes in ambient temperature or calibration drifts [19].

## 3. Results

### 3.1. Case 1: Single Radioisotope

**Figure 2:**
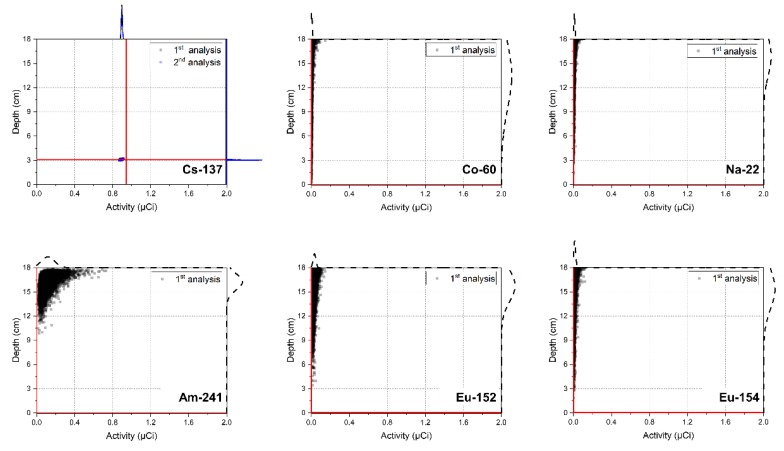
Joint distributions between the depth and activity of the radioisotopes in the radioisotope library for a spectrum acquired for 300 s with a Cs-137 source buried in sand at the depth of 3 cm. The scatter dots represent the correlations between the depth and activity, while red lines and the curves outside the plot area represent their true values and corresponding densities, respectively. The distribution from the first analysis is obscured by that of the second analysis and is hardly visible in the plot for Cs-137.

**Figure 3:**
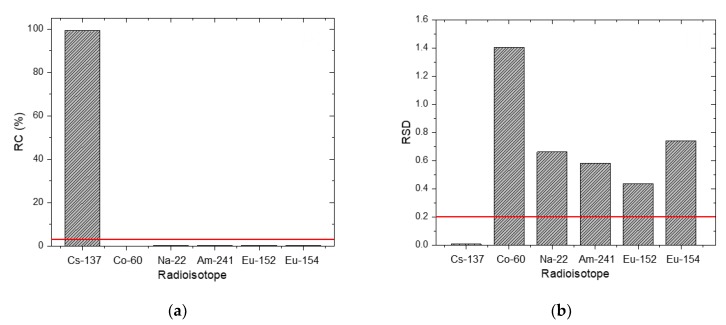
(**a**) RC and (**b**) RSD of the radioisotopes in the radioisotope library for a spectrum acquired for 300 s with a Cs-137 source buried in sand at a depth of 3 cm. The red lines denote criteria for the RC and RSD (i.e., 3% and 0.2, respectively).

Figure 2 shows the joint probability distributions between the depth and activity of the radioisotopes analyzed for a spectrum, measured for 300 s for a Cs-137 source buried at a depth of 3 cm. From the first analysis, we can clearly see that the distribution of the Cs-137 is clustered tightly around the mean, while the distribution of the other radioisotopes (i.e., Co-60, Na-22, Am-241, Eu-152, and Eu-154) is spread along high values of the depth at low activity. As shown in Figure 3, the values of the RCs and RSDs for the five radioisotopes did not satisfy the criteria mentioned in Section 2.3, and therefore only the radioisotope of Cs-137 provided any notable contribution to the spectrum. The second analysis was then performed on the Cs-137 to determine its burial depth and activity. The result confirmed that the joint probability distribution of the Cs-137 was closely centered around the true value of the depth and activity (i.e., 3 cm and 0.942 μCi). It is not always true, however, that the distributed results of the first and second analyses will yield nearly the same output, as we have seen on this occasion. This is because it is possible for this method to induce a distortion in the analysis results by assigning a biased mean of activity to certain radioisotopes during the identification step [25].

**Figure 4:**
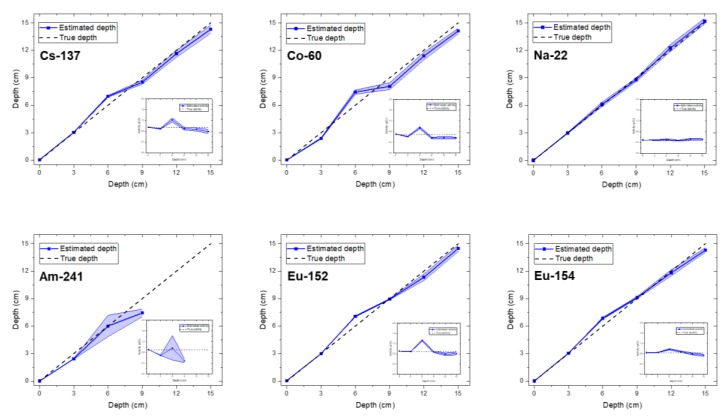
Estimated depth and activity with a 95% credible interval for the single radioisotope of Cs-137, Co-60, Na-22, Am-241, Eu-152, and Eu-154 buried in sand over the range of 0–15 cm at 3 cm intervals. The inset shows the estimated activity of the corresponding radioisotope in each figure.

**Figure 5:**
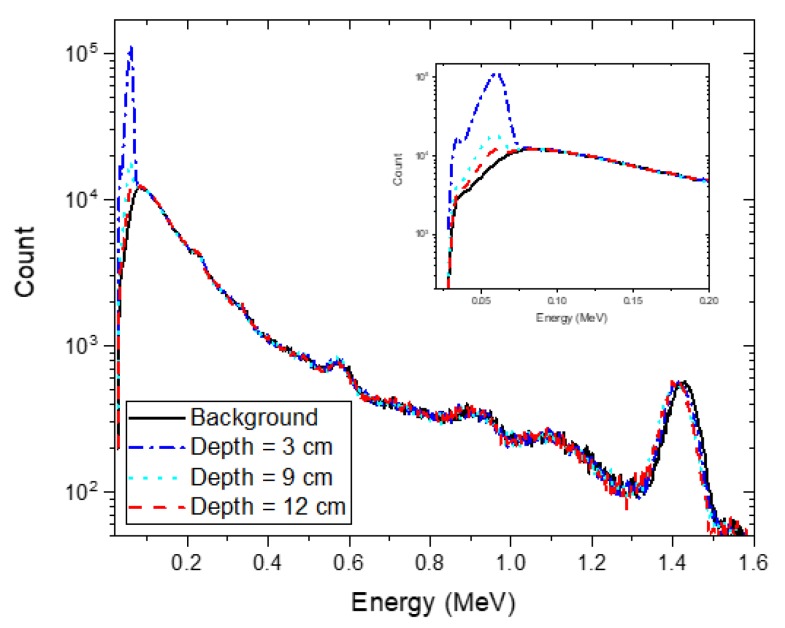
Experimental spectra with an acquisition time of 30 min for an Am-241 source buried in sand at depths of 3 cm (blue dash-dotted line), 9 cm (sky-blue dotted line) and 12 cm (red dashed line). The black solid line represents the background spectrum with the same acquisition time. The inset shows the enlargement of the spectra in the low-energy region.

Figure 4 shows the estimated depth and activity with a 95% credible interval for all single radioisotopes, namely: Cs-137, Co-60, Na-22, Am-241, Eu-152, and Eu-154, buried in sand over a range of 0–15 cm at intervals of 3 cm. The spectra for the analysis were measured for 300 s. From the experimental results, we found that the proposed technique was capable of correctly identifying the buried radioisotopes and determining the depth of the identified radioisotopes with the exception of the Am-241 source at burial depths exceeding 9 cm. At these depths, RSD and RC values for all radioisotopes in the radioisotope library did not meet the criteria, meaning that there were no other radioisotopes affecting the spectra except for the background radiation. This was mainly due to the high attenuation of low-energy photons (e.g., 59 keV) emitted by Am-241. As a consequence, the spectra obtained with the Am-241 source buried deeply became almost indistinguishable from a background spectrum, as shown in Figure 5. Excluding the Am-241, the results confirmed that the true depth was approximated by the mean value of the estimated depth with a 95% credible interval for all radioisotopes with very weak activities that were buried in sand over a range of 0–15 cm; the estimated depths at a depth of 6 cm tend to be slightly higher, probably because of the discrepancy between the measured spectra and the spectra calculated by interpolation. In addition, the estimated mean values of the activity with a 95% credible interval for the identified radioisotopes were in close agreement with the true values. Likewise, the trend in the relationship between the depth and the activity was also in agreement with the results report by Kim et al. [19].

### 3.2. Case 2: Multiple Radioisotopes and Data Acqusition Time

**Figure 6:**
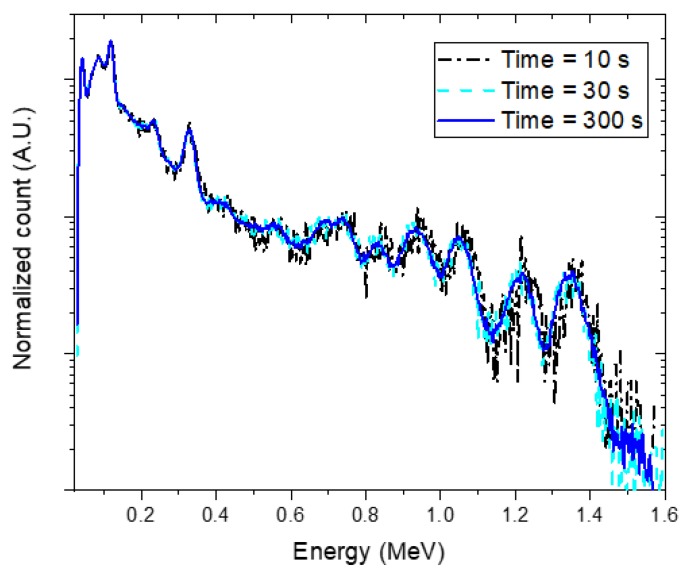
Experimental spectra with different acquisition times of 10 s (black dash-dotted line), 30 s (sky-blue dashed line) and 300 s (blue solid line) for the following radioisotopes buried in sand: Eu-152, 3 cm and Eu-154, 6 cm. Obtained spectra were normalized to the total count over the energies of interest for comparison.

**Figure 7:**
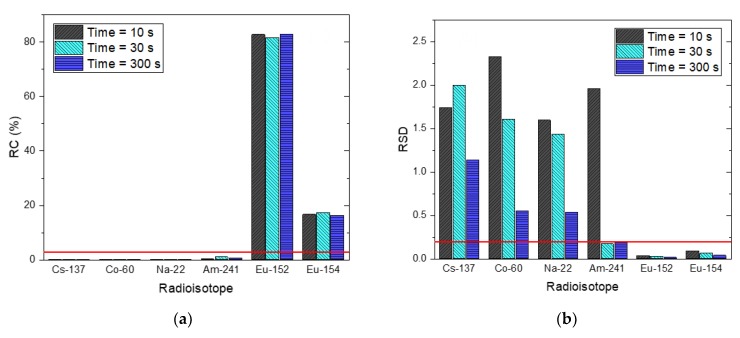
(**a**) RC and (**b**) RSD of radioisotopes in the radioisotope library for three spectra with acquisition times of 10 s, 30 s and 300 s with the following radioisotopes buried in sand: Eu-152, 3 cm and Eu-154, 6 cm. The red lines denote criteria for the RC and RSD (i.e., 3% and 0.2, respectively).

**Figure 8:**
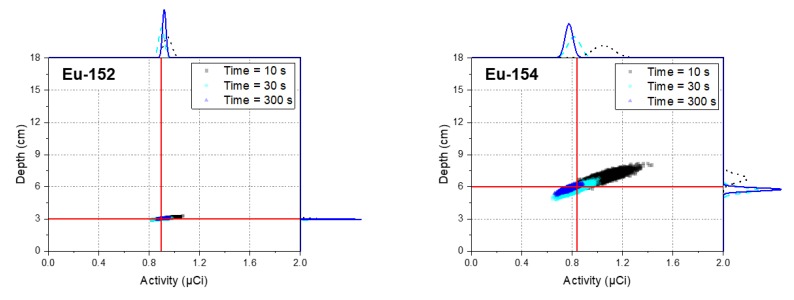
Joint distributions between the depth and activity of identified radioisotopes (i.e., Eu-152 and Eu-154) for experimental spectra acquired with 10 s, 30 s, and 300 s for Eu-152 and Eu-154 sources buried in sand at depths of 3 cm and 6 cm, respectively. The scatter dots represent the correlations between the depth and activity, while the red lines and the curves outside the plot area represent their true values and corresponding densities, respectively.

**Figure 9:**
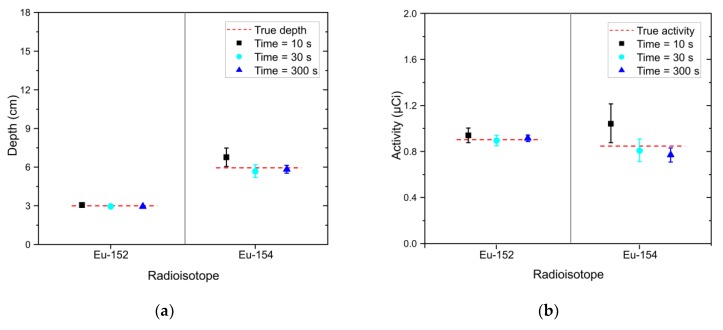
(**a**) Estimated depth and (**b**) activity of identified radioisotopes (i.e., Eu-152 and Eu-154) in the form of mean and 1.96 standard error analyzed for experimental spectra with acquisition times of 10 s (black square), 30 s (sky-blue circle), and 300 s (blue triangle). The spectra were acquired with Eu-152 and Eu-154 sources buried in sand at depths of 3 cm and 6 cm, respectively.

To validate the performance of the proposed method in cases where multiple radioisotopes were buried at different depths, we measured the spectra with different acquisition times of 10 s, 30 s, and 300 s for Eu-152 and Eu-154 sources that were buried in sand at 3 cm and 6 cm in depth, respectively, as shown in Figure 6. The reason for the acquisition of the spectra with the reduced acquisition times was to verify the performance of the proposed method in large-scale field measurements that require a rapid acquisition. As shown in Figure 7, the radioisotopes that had not contributed to the spectra (i.e., Cs-137, Co-60, Na-22, and Am-241) could be rejected in the identification step, meaning that our method can detect the correct radioisotopes for the spectra, even with short acquisition times. The estimated depth and activity of the identified radioisotopes for the spectra are illustrated as joint probability distributions in Figure 8. From these results, the distributions between the depth and activity for the radioisotopes were more closely clustered with increasing acquisition time. In addition, the center of the distributions got closer to the true values. This can be more clearly seen in Figure 9 where the error bar is in the form of mean and 1.96 standard error (or equivalently, a 95% credible interval). This shows that the true values of the depth for the identified radioisotopes indeed fell within the 95% credible interval of the estimated depths analyzed for the spectra. To our surprise, the mean values of the depth analyzed even for the acquisition time of 10 s closely agreed with the true values, which is a much better result than that reported by Kim et al. [19]. This was due to the use of the larger-size more efficient detector. The estimated values of the activity for Eu-154 deviated slightly from the true values. This was possibly due to a position error in the sources during the measurements. In this proposed method, the determination of depth depends primarily on a spectral shape; that is, our approach determines the burial depths of radioisotopes that are most likely to have produced an observed spectrum through the combination of spectral shapes of each radioisotope at varying depths. In contrast, the activity was calculated based mainly on the determined depth and counts in the observed spectrum (see Equation (4)). In this regard, the activity should be accurately estimated once the depths are exactly estimated and the acquisition time is sufficient to reduce the statistical fluctuation present in the observed spectrum.

**Figure 10:**
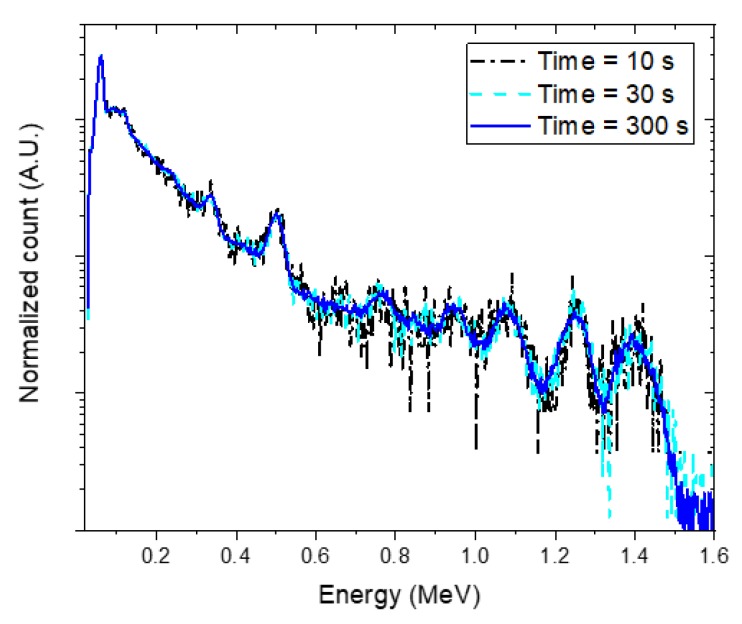
Experimental spectra with different acquisition times of 10 s (black dash-dotted line), 30 s (sky-blue dashed line), and 300 s (blue solid line) for the following radioisotopes buried in sand: Na-22, 10 cm; Am-241, 3 cm; and Eu-152, 8 cm. Obtained spectra were normalized to the total count over the energies of interest for comparison.

**Figure 11:**
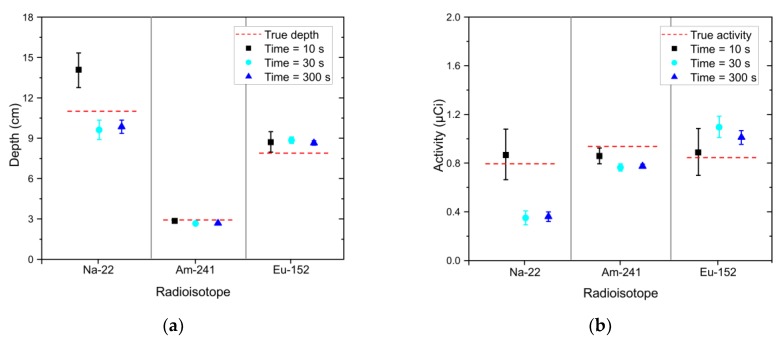
(**a**) Estimated depth and (**b**) activity of identified radioisotopes (i.e., Na-22, Am-241, and Eu-152) in the form of mean and 1.96 standard error analyzed for experimental spectra with acquisition times of 10 s (black square), 30 s (sky-blue circle), and 300 s (blue triangle). The spectra were acquired with Na-22, Am-241, and Eu-152 sources buried in sand at depths of 11 cm, 3 cm, and 8 cm, respectively. The red dotted lines denote the true values.

Figure 10 shows more complex spectra obtained with acquisition times of 10 s, 30 s, and 300 s for Na-22, Am-241, and Eu-152 with burial depths of 10 cm, 3 cm, and 8 cm, respectively. Similar to the previous case, the radioisotopes that are not part of the spectra (i.e., Cs-137, Co-60, and Eu-154) were rejected in the identification step and we could therefore correctly detect the radioisotopes of Na-22, Am-241, and Eu-152. The estimated depth and activity of the identified radioisotopes were reported in terms of mean and 1.96 standard errors, as shown in Figure 11. Here, trends relating to the acquisition time can be observed on the standard errors that are similar to those reported in Figure 9. In particular, the estimated depth of Na-22 for the spectrum with the acquisition time of 10 s showed a relatively large standard error. Also, it deviated from the true value because of the statistical fluctuations in the spectrum. Except this, the results indicated that there was a satisfactory agreement between the estimated and true values for the complex spectrum even where there were the short acquisition times. However, the estimated activities showed relatively more significant deviations from the true values because the activity is inversely related to the square of the depth (see Equation (4)).

### 3.3. Effect of Gain Shift

**Figure 12:**
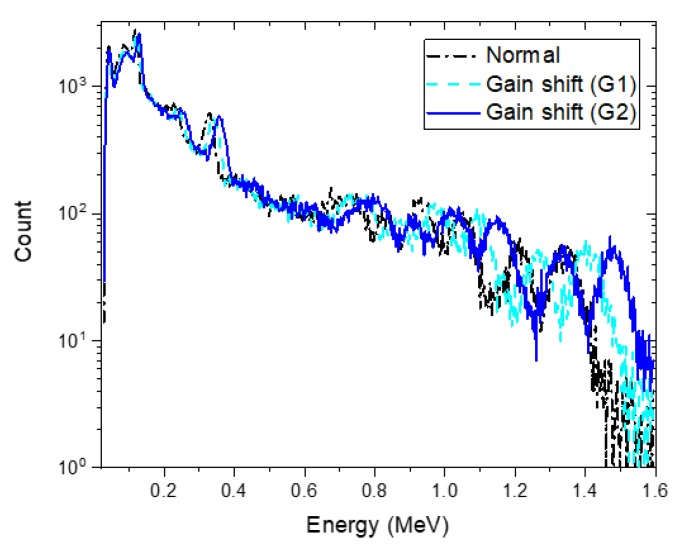
Experimental spectrum composed of Eu-152 and Eu-154 sources buried in sand at depths of 3 cm and 6 cm, respectively (black das-dotted line) and its shifted spectra (sky-blue dashed line and blue solid line) having different magnitudes of the shift due to calibration drifts. The spectra were acquired for 30 s.

**Figure 13:**
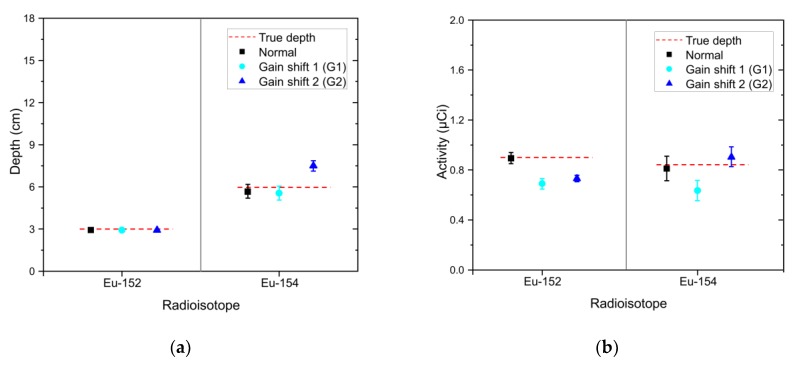
(**a**) Estimated depth and (**b**) activity of identified radioisotopes (i.e., Eu-152 and Eu-154) in the form of mean and 1.96 standard error analyzed for a 30-s measured spectrum composed of Eu-152 and Eu-154 buried in sand at depths of 3 cm and 6 cm (black square) and its shifted spectra (sky-blue circle and blue triangle). The red dotted lines denote the true values.

To investigate how well the proposed technique would accurately analyze shifted spectra because of gain-shift effects, we acquired spectra at an acquisition time of 30 s for the Eu-152 and Eu-154 sources at burial depths of 3 cm and 6 cm after calibration drifts had occurred in the detector. Figure 12 shows these spectra with two different magnitudes of the shift (blue-sky dotted line and blue solid line referred to as “G1” and “G2”) and the normal spectrum (black dash-dotted line) for comparison. In fact, these shifted spectra would be very difficult to analyze without proper recalibration settings; the position of the original photo-peak in the high-energy region of the normal spectrum was overlapped completely by another peak in the G2 spectrum. Nevertheless, the proposed method was able to exclude the radioisotopes that had not contributed to the shifted spectra in the identification step. For the G1 spectrum, the estimated depths of Eu-152 and Eu-154 closely agree with the true values as shown in Figure 13a. For the G2 spectrum, the mean value of the estimated depth for Eu-154 was 7.52 ± 0.38 (1.96σ) cm, which was slightly overestimated. The activity tended to be underestimated against the determined depths as the spectrum shifted in a positive direction (see Figure 13b). This was possibly due to an increase in full width at half maximum as the spectrum moves to higher energies, resulting in a reduction in the maximum counts in the region of the photo-peaks, which does not occur in spectra that were shifted mathematically via interpolation. Overall, the presented results demonstrated a capability to accommodate a shift in the spectra caused by calibration drift in the complex spectra of multiple radioisotopes.

## 4. Discussion

In this work, we demonstrated the estimation of remote depth profiling for contaminated low-level radioactive materials that are composed of single or multiple radioisotopes by applying Bayesian inference. An earlier report had already shown that this approach is reliable and robust because it allows us to offer the mean and standard error for the estimated depth and activity from a single measurement [19]. Also, the reported experimental results demonstrated that significant improvements in earlier findings had been achieved. First, the proposed technique does not rely on an assumption that we have foreknowledge of a radioisotope present at the site, or that only a single radioisotope exists there. Instead, this method first identifies unknown radioisotopes and then determines the depth and activity of the identified source(s). Thus, we are not only able to identify individual radioisotopes for spectra composed of multiple radioisotopes, but also to provide a good approximation of each one’s depth and activity. Second, the results showed that this method can be applied to both low-level buried wastes and all gamma-emitting radioisotopes, regardless of the intensity of the gamma-ray energy or the number of gamma rays emitted, given that photons are not fully attenuated in a substance and contribute to the spectra to some degree. Lastly, we demonstrated that this method is also capable of accommodating the gain-shift effects in spectra with multiple radioisotopes.

One of the challenges associated with the measurement point is that it is difficult to find an optimal position for the detector in relation to the location of the buried radioactive contaminants. An alternative solution could be to position the detector at the location with the maximum intensity of total count rate. However, multiple contaminants buried at different depths may not be vertically positioned. If they are located in that way, the error of the *x-y* position causes an error of the burial depth (*z* position). Further work must be conducted to resolve this issue so that a better approximation of localized radioactive wastes in three dimensions can be achieved.

## 5. Conclusions

In this work, we presented a novel method for the remote depth estimation of unknown radioactive contaminants using Bayesian inference. Experimental results confirmed that this method correctly identifies radioactive contaminants composed of multiple radioisotopes as well as a single radioisotope and provides good estimates of depths buried in sand for the identified isotopes in a single measurement. In addition, we demonstrated that short acquisition time and gain-shift effects did not significantly degrade the analysis results for spectra composed of multiple radioisotopes. These results showed significantly improved remote depth estimation capability in comparison with the existing methods. Therefore, the proposed method is capable of achieving a rapid nonintrusive localization of buried low-level multiple radioactive contaminants through in situ measurement.

## References

[1] Characterization of Radioactively Contaminated Sites for Remediation Purposes. https://www-pub.iaea.org/MTCD/publications/PDF/te_1017_prn.pdf.

[2] Radiological Characterisation for Decommissioning of Nuclear Installations. https://www.oecd-nea.org/rwm/docs/2013/rwm-wpdd2013-2.pdf.

[3] Multi-Agency Radiation Survey and Site Investigation Manual (MARSSIM). (NUREG-1575, Revision 1). https://www.nrc.gov/reading-rm/doc-collections/nuregs/staff/sr1575/r1/.

[4] Sullivan P.O., Nokhamzon J.G., Cantrel E. (2010). Decontamination and Dismantling of Radioactive Concrete Structures. NEA News.

[5] Dennis F., Morgan G., Henderson F. (2007). Dounreay Hot Particles: The Story so Far. J. Radiol. Prot..

[6] Dounreay Particles Advisory Group. https://assets.publishing.service.gov.uk/government/uploads/system/uploads/attachment_data/file/696380/DPAG_3rd__Report_September_2006.pdf.

[7] Popp A., Ardouin C., Alexander M., Blackley R., Murray A. Improvement of a High Risk Category Source Buried in the Grounds of a Hospital in Cambodia. Proceedings of the 3th International Congress of the International Radiation Protection Association (IRPA).

[8] Control of Orphan Sources and Other Radioactive Material in the Metal Recycling and Production Industries. https://www-pub.iaea.org/MTCD/Publications/PDF/Pub1509_web.pdf.

[9] Maeda K., Sasaki S., Kumai M., Sato I., Suto M., Ohsaka M., Goto T., Sakai H., Chigira T., Murata H. (2014). Distribution of Radioactive Nuclides of Boring Core Samples Extracted from Concrete Structures of Reactor Buildings in the Fukushima Daiichi Nuclear Power Plant. J. Nucl. Sci. Technol..

[10] Shippen B.A., Joyce M.J. (2011). Extension of the Linear Depth Attenuation Method for the Radioactivity Depth Analysis Tool (RADPAT). IEEE Trans. Nucl. Sci..

[11] Shippen A., Joyce M.J. (2010). Profiling the Depth of Caesium-137 Contamination in Concrete via a Relative Linear Attenuation Model. Appl. Radiat. Isot..

[12] Adams J.C., Joyce M.J., Mellor M. (2012). The Advancement of a Technique Using Principal Component Analysis for the Non-Intrusive Depth Profiling of Radioactive Contamination. IEEE Trans. Nucl. Sci..

[13] Adams J.C., Joyce M.J., Mellor M. (2012). Depth Profiling 137Cs and 60Co Non-Intrusively for a Suite of Industrial Shielding Materials and at Depths beyond 50mm. Appl. Radiat. Isot..

[14] Adams J.C., Mellor M., Joyce M.J. (2011). Determination of the Depth of Localized Radioactive Contamination by 137Cs and 60Co in Sand with Principal Component Analysis. Environ. Sci. Technol..

[15] Ukaegbu I.K., Gamage K.A.A. (2018). A Novel Method for Remote Depth Estimation of Buried Radioactive Contamination. Sensors.

[16] Ukaegbu I.K., Gamage K.A.A. (2018). A Model for Remote Depth Estimation of Buried Radioactive Wastes Using CdZnTe Detector. Sensors.

[17] Ukaegbu I.K., Gamage K.A.A., Aspinall M.D. (2019). Nonintrusive Depth Estimation of Buried Radioactive Wastes Using Ground Penetrating Radar and a Gamma Ray Detector. Remote Sens..

[18] Adams J.C., Mellor M., Joyce M.J. (2010). Depth Determination of Buried Caesium-137 and Cobalt-60 Sources Using Scatter Peak Data. IEEE Trans. Nucl. Sci..

[19] Kim J., Lim K.T., Park K., Cho G. (2019). A Bayesian Approach for Remote Depth Estimation of Buried Low-Level Radioactive Waste with a NaI (Tl) Detector. Sensors.

[20] Wagenmakers E.-J., Lee M., Lodewyckx T., Iverson G.J. (2008). Bayesian Versus Frequentist Inference. Bayesian Evaluation of Informative Hypotheses.

[21] Philosophy of Statistics. https://plato.stanford.edu/entries/statistics/#pagetopright.

[22] Andrieu C., Freitas N., Doucet A., Jordan M.I. (2003). An Introduction to MCMC for Machine Learning. Mach. Learn..

[23] Mullachery V., Khera A., Husain A. (2018). Bayesian Neural Networks. arXiv.

[24] Kucukelbir A., Tran D., Gelman A., Blei D.M. (2017). Automatic Differentiation Variational Inference. J. Mach. Learn. Res..

[25] Kim J., Taek K., Kim J., Kim Y., Kim H. (2019). Quantification and Uncertainty Analysis of Low-Resolution Gamma-Ray Spectrometry Using Bayesian Inference. Nucl. Instrum. Methods Phys. Res. Sect. A Accel. Spectrom. Detect. Assoc. Equip..

